# The role of esophageal area for dysphagia after anterior cervical corpectomy fusion: Change trends and risk factors

**DOI:** 10.1097/MD.0000000000032974

**Published:** 2023-02-17

**Authors:** Yanyan Ma, Peiming Sang, Binhui Chen

**Affiliations:** a Department of Gastroenterology, Lihuili Hospital of Ningbo Medical Center, Ningbo, Zhejiang, PR China; b Department of Orthopedic Surgery, Lihuili Hospital of Ningbo Medical Center, Ningbo, Zhejiang, PR China.

**Keywords:** anterior cervical corpectomy fusion, cervical spine, dysphagia, esophageal area

## Abstract

The objective of this study is to assess the change trends of perioperative esophageal area for anterior cervical corpectomy fusion (ACCF) and to analyze the risk factors of the area for postoperative dysphagia. We retrospectively analyzed 309 patients who underwent ACCF due to degenerative cervical diseases between November 2015 and September 2019 at our hospital. Patients were divided into 2 groups named the dysphagia group and the normal swallowing function group, according to the swallowing function after ACCF. The esophageal area was measured at T1 level using computed tomography axial plane images before and after surgery (1 week, 1 month, 8 months, and 12 months), in order to assess the change trends of esophageal area perioperatively and analyze risk factors of the area for dysphagia after ACCF. The area was highest at 1 week after surgery and would be decreased over time in both groups, which was recovered to the preoperative levels in 12 months after surgery. The incidence of dysphagia after ACCF was 41.1%. In the dysphagia group, 127 patients (mean age 59.299 years) had dysphagia after ACCF. In the normal-swallowing function group, 182 patients (mean age 59.8352 years) had normal swallowing function after ACCF. The preoperative esophageal area was larger in the dysphagia group than in the normal-swallowing function group. Preoperative esophageal area was correlated with postoperative dysphagia (odds ratio: 1.3457, 95% confidence interval: 1.106–1.637). When the esophageal area at preoperation was above 3.388 cm^2^, the risk of postoperative dysphagia was higher. The esophageal area was the biggest at 1 week postoperatively, significantly decreased over time and would be recovered to the normal size at 12 months after surgery. Preoperative esophageal area should be considered when evaluating the risk factor for dysphagia after ACCF.

## 1. Introduction

Anterior cervical corpectomy fusion (ACCF) is used to treat cervical disorders. The esophagus must be pulled to the contralateral position to improve operative field exposure during anterior cervical spinal surgery. The esophagus swells due to the local ischemia of the esophageal wall caused by retractor blades, which may give rise to postoperative dysphagia. Postoperative dysphagia is common.^[1]^ Several studies reported the incidence of dysphagia after surgery between 2% and 60%; this wide range was likely due to the various methods of assessing dysphagia and differences in study design.^[2–8]^ Prevertebral soft tissue swelling after anterior cervical spinal surgery has been described. Sanfilippo et al found that prevertebral soft tissue swelling after anterior cervical spinal surgery lasts 2 weeks and decreases after 6 weeks.^[9]^

Few study has reported the change of esophageal area for dysphagia after ACCF or analyzed their relationship between them. Therefore, the present study calculated the correlation between esophageal area and dysphagia. This calculation allowed the prediction of the risk of postoperative dysphagia, in order to take some actions to prevent if the risk was higher.

## 2. Materials and methods

We considered 309 patients who underwent ACCF due to degenerative cervical diseases in our hospital between November 2015 and September 2019. The esophageal area was determined by measuring the esophageal area in the level of T1 using computed tomography axial plane images. The Hospital’s Research Ethics Board approved the study (Number: 2017025, Date: December 23, 2017).

### 2.1. Surgical method

ACCF was performed under general anesthesia and approached from the right side. A segmental vertebra, material from 2 adjacent discs, endplate cartilage, and posterior longitudinal ligament were removed to achieve complete decompression. Appropriate titanium mesh (Stryker Spine S.A.S, VBOSS, France) filled with autologous cancellous bone was inserted into the intervertebral space, and a plate was fixed (Aesculap, ABC, Germany, or MedioxOrvosiMuszergyartoKft, Anterior Spinal Plate, Hungary). A closed suction drain was used, and the drain was removed on the second day after surgery.

**Figure 1.**
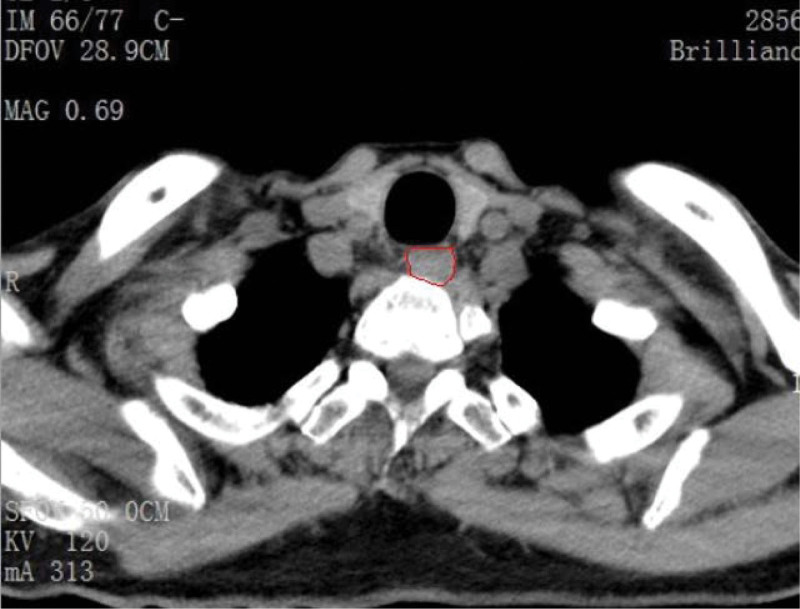
Esophageal area was measured in the level of T1 using CT axial plane images with Software of Digimizer Image Analysis. CT = computed tomography.

We evaluated swallowing quality at 1 week, 1 month, 8 months, and 12 months after ACCF according to the standard of the Swallowing Quality of Life.^[1,10]^ Chief complaints, physical examinations, and radiological exams, including X-ray, computed tomography (CT), and magnetic resonance imaging, were tested preoperatively. After surgery, cervical spinal column CT and X-ray were performed at 1 week, 1 month, 8 months, and 12 months. The esophageal area was measured in the level of T1 using CT axial plane images with Software of Digimizer Image Analysis (Fig. 1).

### 2.2. Statistical analysis

Statistical analysis was performed using SPSS ver. 16.0 (SPSS Inc., Chicago, IL). The analysis of variance for repeated measurement data and the least-squares difference test were used to determine significant differences in the esophageal area at the different periods. *P* < .05 indicated significant differences. Logistic regression models were created to assess the risk factors of esophageal swelling perioperatively for dysphagia after ACCF. Receiver operating characteristic curves were used to determine optimal threshold values of esophageal area for dysphagia after ACCF.

## 3. Results

There were 309 patients divided into 2 groups according to swallowing function after ACCF. In the dysphagia group, there were 79 men and 48 women with a mean age of 59.299 years. In the normal-swallowing function group, there were 131 men and 51 women with a mean age of 59.8352 years. There were not significant differences about gender and age between the 2 groups, which were shown at Table 1.

**Table 1:** Patient characteristics by group.

	Dysphagia group	Normal- swallowing group	*P* value
Number of patients	127	182	
Gender			
Male	79	131	
Female	48	51	.082875
Age	59.299 ± 10.227	59.8352 ± 8.24723	.61132

The incidence of dysphagia after ACCF was 41.1%. In the dysphagia group, 120 of 127 (94.5%) patients had dysphagia that resolved 8 months after ACCF, and all resolved by 12 months. The esophageal areas were displayed in Table 2.

**Table 2:** Esophageal area at different period in 2 groups (cm^2^).

	The dysphagia group	The normal-swallowing group
Preoperation	3.0 ± 1.302	2.573 ± 1.149
1 wk after operation	5.07 ± 2.11*	4.797 ± 1.398¶
1 mo after operation	4.04 ± 1.85*†	3.834 ± 1.08¶#
8 mo after operation	3.197 ± 1.36†‡∥	2.985 ± 1.017¶#**
12 mo after operation	3.047 ± 1.298†‡§∥	2.59 ± 1.1264#**††

* *P* < .05 versus pre.

† *P* < .05 versus 1 wk.

‡ *P* < .05 versus 1 mo.

§ *P* > .05 versus 8 mo.

∥ *P* > .05 versus pre.

¶ *P* < .05 versus pre.

# *P* < .05 versus 1 wk.

** *P* < .05 versus 1 mo.

†† *P* > .05 versus pre.

In the dysphagia group, the esophageal areas were the highest at 1 week and significantly decreased from 1 week to 8 months. Esophageal areas at 8 and 12 months postoperatively were equal to the preoperative sizes.

In the normal swallowing group, there were 182 patients who underwent ACCF had normal-swallowing function. The esophageal areas were displayed in Table 2. The areas were the largest at 1 week postoperatively and decreased slowly from 1 week to 12 months (all *P* < .05) in the normal-swallowing group. There was no significant change between preoperative measurements and 12 months postoperatively.

The value of perioperative esophageal area was compared between the 2 groups, which was shown at Table 3. It was the significant difference for the preoperative esophageal area between the 2 groups. Although there were different about the postoperative esophageal area and the postoperative change, the differences were not significant statistically.

**Table 3:** The value of perioperative esophageal area in 2 groups (cm^2^).

	The dysphagia group	The normal-swallowing group	*P* value
Pre operation	3.0 ± 1.302	2.573 ± 1.149	.002527
1 wk after operation	5.07 ± 2.11	0.1708485	4.797 ± 1.398
The change at 1 wk postoperatively	2.2238 ± 1.5369	2.0699 ± 1.66438	.40323

a < 0.05 vs dysphagia group in preoperation. b > 0.05 vs dysphagia group in 1week after operation. c > 0.05 vs dysphagia group for the change of esophageal area at 1week postoperatively.

**Figure 2.**
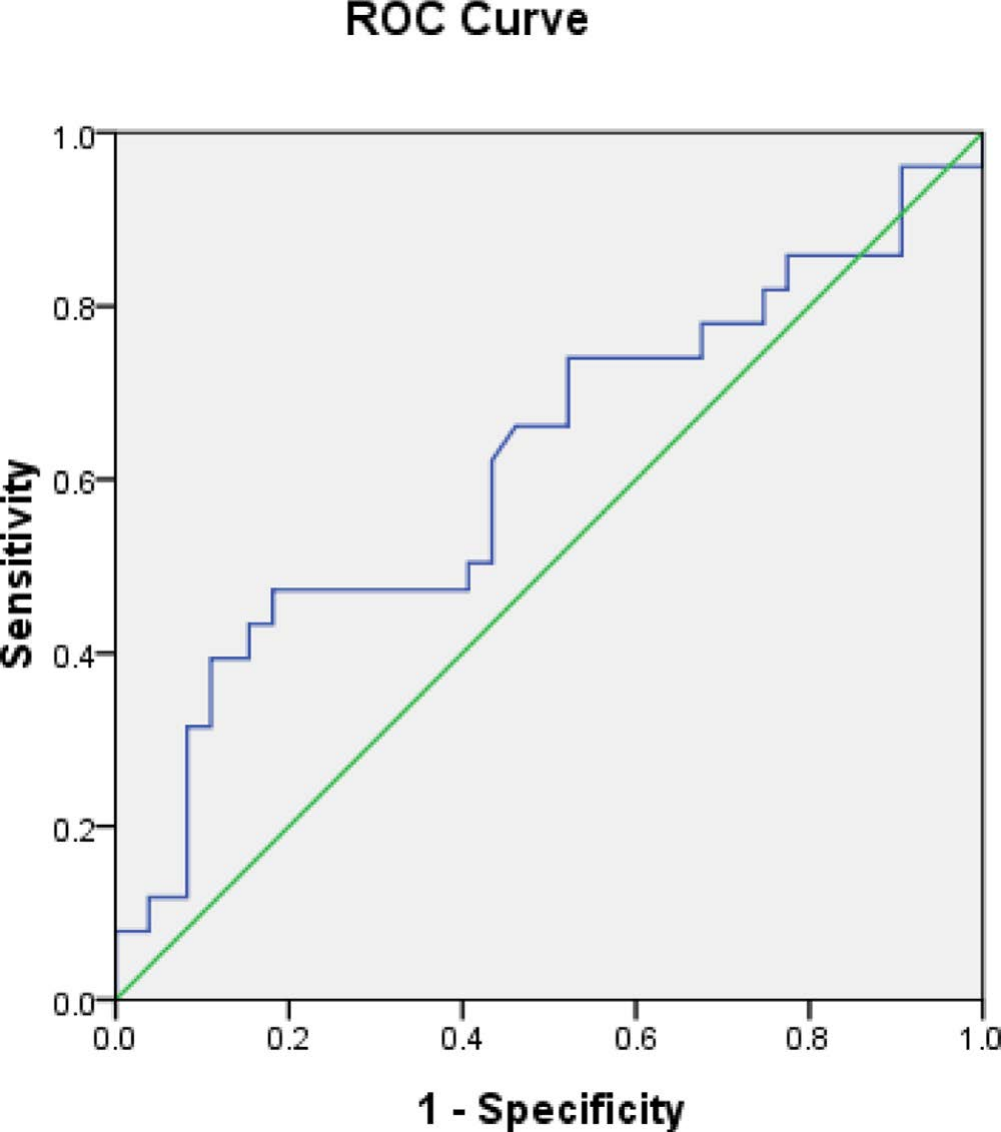
ROC analysis of preoperative esophageal area. ROC = receiver operating characteristic.

To determine risk factors of dysphagia following ACCF, we measured the preoperative esophageal area using a logistic regression model (Table 4) and found that preoperative esophageal area was correlated with postoperative dysphagia (odds ratio: 1.3457, 95% confidence interval: 1.106-1.637). Receiver operating characteristic analysis of preoperative esophageal area was shown in Figure 2. The cutoff value for preoperative esophageal area ≦3.388 cm^2^.

**Table 4:** Logistic regression for esophageal area of dysphagia during ACCF.

Period	*B*	S.E.	Wald	df	Sig.	OR	95% CI of OR	
							Lower	Upper
Preoperation	0.2969	0.1	8.803	1	0.003	1.3457	1.106	1.637
Constant	−1.1897	0.308	14.915	1	1.124 × 10^−4^	0.3043		

ACCF = anterior cervical corpectomy fusion, CI = confidence interval, OR = odds ratio.

## 4. Discussion

Dysphagia is a common complication after anterior cervical spinal surgery. Riley et al reported that the rates of dysphagia after anterior cervical spinal surgery ranged from 1% to 79% within a week of surgery, decreasing to 50% to 56% after a month, and reaching a plateau at 1 year, when the rates ranged between 13% and 21%.^[11]^ These findings agreed with our result in the present study, which the rate of postoperative dysphagia was 41.1% in our study.

It was very significant to assess the change trends of esophageal area after ACCF. Soft tissue swelling after anterior cervical spinal surgery occurs in almost all patients. Many studies assessed soft tissue swelling after anterior cervical surgery by measuring the widths of prevertebral soft tissues. A considerable amount of literature described the role of prevertebral soft tissue after anterior cervical spinal surgery.^[12–15]^ These studies showed prevertebral soft tissue swelling after anterior cervical spinal surgery. Sanfilippo et al reported that prevertebral soft tissue swelling after anterior cervical spinal surgery lasted for 2 weeks and decreased after 6 weeks, this swelling dissipated to normal or near-normal levels by the 6-week follow-up.^[9]^ According to the measurement of perioperative esophageal area in our study, the esophageal swelling were the biggest at 1 week postoperatively, significantly decreased over time and would be recovered to the normal level at 12 months after surgery. So it was more important for our doctors to care about the patients^’^ esophageal swelling and swallowing function in the short time after surgery. If the esophageal area has been increased persistently for a long time after surgery, the infection and esophageal perforation should be considered, in which some tests must be done and the actions should be taken to prevent or treat as soon as possible. It is very fatal for the patients when the infection or esophageal perforation happens.

Prevertebral soft tissue swelling after anterior cervical spine surgery was related to postoperative dysphagia. Studies showed that the postoperative dysphagia was related to intraoperative retraction of the pharyngeal/esophageal wall that caused higher pressure on the esophagus and lower perfusion to the adjacent tissue.^[16,17]^ During the surgery, local ischemia of the pharyngeal/esophageal wall from retractor blades caused reperfusion trauma, edema and swelling, which could subsequently result in early postoperative dysphagia. Martin et al showed that increased prevertebral soft tissue might contribute to dysphagia after anterior cervical spine surgery.^[4]^ Yamagata et al found that patients with C3 prevertebral soft tissue swollen at 2 or 3 days after surgery were 8.7 times more likely to have postoperative dysphagia.^[18]^ Patients with C4 prevertebral soft tissue swelling at 2 or 3 days after surgery were 4.7 times more likely to have postoperative dysphagia. Yagi et al found that prevertebral soft tissue edema was related to severe dysphagia and odynophagia after anterior cervical discectomy and fusion.^[19]^ Studies showed that prevertebral soft tissue edema was associated with postoperative dysphagia that peaked at 3 to 4 days after anterior cervical spinal surgery, prevertebral soft tissue edema was considered as tissue damage.^[20,21]^ Other studies suggested the intermittent release of retractors to minimize the injury of compression-associated tissue ischemia.^[16,22]^

Due to the approach of anterior cervical spine surgery, the internal organs, including the esophageal, must be retracted to the contralateral position intraoperatively, which caused compression-associated tissue ischemia, the esophageal swelling and postoperative dysphagia. With the extended follow-up time, the esophageal swelling would be reduced because of the peristalsis of esophageal by itself, which swallowing function would be recovered to the normal. So according to this present regulation, it was more significant for the patients with postoperative dysphagia to get the decreasing trend of esophageal area in the short time after surgery. Only in this way, the swallowing function would be returned to the normal in the future.

In our study, the preoperative esophageal area was bigger in the dysphagia group than in the normal-swallowing group, and the area was a significant risk factor for predicting postoperative dysphagia. The cutoff threshold for the preoperative esophageal area was ≦3.388 cm^2^. Before anterior cervical corpectomy surgery, it was essential to predict the risk of postoperative dysphagia according to the value of preoperative esophageal area. If the preoperative esophageal area >3.388 cm^2^, the risk of the postoperative dysphagia was higher, which the doctors and patients should know before surgery. That would avoid patients’ dispute about postoperative dysphagia.

If the risk of postoperative dysphagia is higher, some preventive actions should be taken. Liu et al stated that adequate preoperative preparation should include preoperative tracheal traction exercise and smoking cessation, preventive measures during surgery should include endotracheal tube cuff pressure of 20 mm Hg, avoiding routine use of rhBMP-2, use of a zero-profile implant, use of Zephir plate, use of a new cervical retractor, steroid treatment, avoidance of prolonged operating time, avoidance of overenlargement of cervical lordosis, and decreasing the number of surgical levels.^[23]^ Physicians should fortify their knowledge of anatomy to improve surgical and comfort techniques that are essential to prevent early and persistent dysphagia after anterior cervical spine surgery.

### 4.1. Limitations

This study has some significant limitations. First, the sample size was relatively small, and the follow-up period was relatively short, reducing the accuracy. Second, it was a retrospective study. Third, the effect of preventive actions for the dysphagia was not verified in our study. Further research would be needed.

## 5. Conclusions

The esophagus swells following surgery, causing dysphagia. The esophageal swelling was the biggest at 1 week postoperatively, significantly decreased over time and would be recovered to the normal size at 12 months after surgery. The incidence of postoperative dysphagia decreases with the resolution of esophageal swelling over time. Preoperative esophageal area should be considered as a risk of postoperative dysphagia. The cutoff threshold for the preoperative esophageal area was ≦3.388 cm^2^. when the preoperative esophageal area > 3.388 cm^2^, the risk of the postoperative dysphagia was higher and some preventive actions should be taken perioperatively, which would avoid occurrence of postoperative dysphagia.

## Author contributions

**Data curation:** Yanyan Ma, Peiming Sang, Binhui Chen.

**Formal analysis:** Yanyan Ma, Peiming Sang.

**Writing – original draft:** Yanyan Ma.

**Writing – review & editing:** Yanyan Ma.
